# Conformations of Islet Amyloid Polypeptide Monomers in a Membrane Environment: Implications for Fibril Formation

**DOI:** 10.1371/journal.pone.0047150

**Published:** 2012-11-02

**Authors:** Mojie Duan, Jue Fan, Shuanghong Huo

**Affiliations:** Gustaf H. Carlson School of Chemistry and Biochemistry, Clark University, Worcester, Massachusetts, United States of America; University of Maryland, United States of America

## Abstract

The amyloid fibrils formed by islet amyloid polypeptide (IAPP) are associated with type II diabetes. One of the proposed mechanisms of the toxicity of IAPP is that it causes membrane damage. The fatal mutation of S20G human IAPP was reported to lead to early onset of type II diabetes and high tendency of amyloid formation *in vitro*. Characterizing the structural features of the S20G mutant in its monomeric state is experimentally difficult because of its unusually fast aggregation rate. Computational work complements experimental studies. We performed a series of molecular dynamics simulations of the monomeric state of human variants in the membrane. Our simulations are validated by extensive comparisons with experimental data. We find that a helical disruption at His18 is common to both human variants. An L-shaped motif of S20G mutant is observed in one of the conformational families. This motif that bends at His18 resembles the overall topology of IAPP fibrils. The conformational preorganization into the fibril-like topology provides a possible explanation for the fast aggregation rate of S20G IAPP.

## Introduction

The amyloid fibrils found in post-mortem individuals with type II diabetes are mainly composed of islet amyloid polypeptide (IAPP), also known as amylin [1], [2], [3]. IAPP is a peptide hormone that is co-secreted with insulin by the islet β-cells of the pancreas. A single mutation of serine-to-glycine at position 20 is associated with early onset of type II diabetes [4], [5] and enhanced amyloid formation, reflected in larger quantity of fibrils and faster rate of fibrillation than the wild type [6], [7], [8]. Although the native structure of human IAPP (hIAPP) is disordered, experiments combined with simulations have uncovered several distinct conformational families of hIAPP monomers, including α-helical conformations [9], [10], [11]. The transient helical structure is stabilized in membrane environments demonstrated by the NMR data obtained under a range of conditions [12], [13].

One of the proposed mechanisms of hIAPP cytotoxicity is that hIAPP induces membrane damage to the β cells [14], [15]. However, models on how hIAPP permeabilizes membranes are mutually exclusive. A “pore-like” model suggests that the hIAPP oligomers form transmembrane pore structures [16], [17], [18], [19], while recent experiments indicate that the growth of hIAPP fibrils at the membrane is the cause of membrane leakage [20], [21]. The seemingly different results *in vitro* may be due to the differences in membrane composition, buffer conditions (i.e. ionic strength, pH, and the types of salts), and the process of the preparation of hIAPP solutions [22], [23].

To elucidate the mechanism of hIAPP cytotoxicity, a significant amount of effort has been made to study IAPP-membrane interactions [14], [22], [24], [25]. Site-directed spin labeling and EPR spectroscopy were used to investigate the monomeric form of hIAPP in large unilamellar vesicles containing 80% 1-palmitoyl-2-oleoyl-sn-glycero-3-phospho-L-serine and 20% 1-palmitoyl-2-oleoyl-sn-glycero-3-phosphocholine [26]. The helical periodicity shows that residues 9–22 of hIAPP form an α-helix that adopts a parallel orientation with respect to the membrane surface. The center of the helix immerses into the membrane by 6–9 Å below the level of the phosphate groups. A helical conformation was also detected in a similar region for rat IAPP in micelles [27]. Membrane environment accelerates hIAPP fibril formation [28], [29], [30]. It was proposed that the formation of helical structures is the intermediate step towards hIAPP amyloid assembly in membranes [31], [32], [33]. The goal of this work is to investigate the initial stage of IAPP-membrane interaction and the effect of S20G mutation on the helical structure in the membrane environment using molecular dynamics simulations. Characterizing the structural features of this mutant in its monomeric state is experimentally difficult because of its fast aggregation rate [8]. Computational approaches that provide structural information at the atomic level complement experimental studies. Our simulations provide insights into how the conformational differences between the wild type and the mutant in the monomeric state may affect the later amyloid formation.

## Materials and Methods

### Membrane Construction

In order to mimic the membrane environment of the β-cells, we construct the membrane using 1,2-dioleoyl-sn-glycero-3-phosphocholine (DOPC)/1,2-dioleoyl-sn-glycero-3-phospho-L-serine (DOPS) in a roughly 7∶3 molar ratio. This construction reflects the observed ratio of neutral to anionic phospholipids within β-cells [34]. The previously equilibrated coordinates of 128 DOPC lipids and 128 DOPS lipids as well as the corresponding topology files were obtained from the work of Polyansky *et al.* [35]. Forty DOPC lipids were then randomly replaced by the DOPS lipids, resulted in 40 DOPS lipids and 88 DOPC lipids. Because DOPS bears −1 charge, 40 Na^+^ ions were added to neutralize the system. The lipid/counter ion system was solvated with 5528 SPC (simple point charge) water molecules, resulted in nearly 24,000 atoms in a box of 64 Å×64 Å×80 Å.

### Simulation Protocols of the DOPC/DOPS Bilayer

The system was subjected to the steepest descent energy minimization to remove unfavorable contacts. The minimization stopped when the maximum force <1000.0 kJ/mol⋅nm or the minimization steps reached 50000 steps, whichever came first. The GROMACS package (version 4.5.1) [36] was used for the minimization and the subsequent molecular dynamics (MD). To equilibrate the system, we performed an MD run in the NVT ensemble for 50 ps followed by an additional 1-ns MD run in the NPT ensemble. Finally, a 50-ns MD production run was carried out in the NPT ensemble. The 3D periodic boundary conditions with a 2-fs time step were used in the simulation. The Parrinello-Rahman [37] pressure coupling method was employed to maintain an isotropic constant pressure of 1.0 bar with a coupling time constant of 2.0 ps. The temperature was kept constant at 300 K using the Berendsen thermostat [38] for the NVT run and the Nosé-Hoover thermostat [39], [40] was used for the NPT runs with a coupling time constant of 0.1 ps. The snapshots were saved every 2 ps. All bonds were constrained with the LINCS algorithm [41]. The electrostatic interactions were treated with the particle-mesh Ewald summation [42], [43], and a 12-Å cutoff was used for the van der Waals interactions.

### Peptide Fragments

Lines of experimental evidence have shown that IAPP interacts with the membranes by its N-terminal segment [12], [13], [26], [30]. The amino acid range that forms the initial intermolecular contact in aggregation was determined to be residues 11–25 by the limited proteolysis [44]. Therefore, the fragment of residue 1 to 25 was studied in this work. A series of MD simulations was performed on the membrane-bound IAPP fragment with a charged N-terminus and an amidated C-terminus. The fragments are wild-type hIAPP_1–25_ with neutral His18 (protonated on Nd1) for a total charge of +3, S20G hIAPP_1–25_ with neutral His18 (protonated on Nd1) for a total charge of +3, and wild-type hIAPP_1–25_ with doubly protonated His18 for a total charge of +4.

### Initial Structure and Orientation of the Peptides

Although the consensus in the literature is that the N-terminal segment is a helical structure, the length of the helical region varies [13], [26], [27], [30]. The reported longest helical region spans residues 5–28 [13]. We built residue 5 to 25 into an α-helix, which is the longest possible helix for the fragment in our study. The disulfide bridge between Cys2 and Cys7 makes the first four residues form a disordered hairpin loop. The initial orientation of the IAPP peptides relative to the membrane surface is based on the data of EPR spectroscopy of spin-labeled hIAPP derivatives [26]. The α-helical region of IAPP is parallel to the membrane surface with Thr9, Leu12, Leu16 and Ser20 buried inside the membrane and Gln10, Asn14, and His18 exposed to the solvent. The center of the helix is immersed into the membrane for 7 Å below the average position of the phosphate groups.

### Simulations of the Peptide-membrane Systems

**Figure 1:**
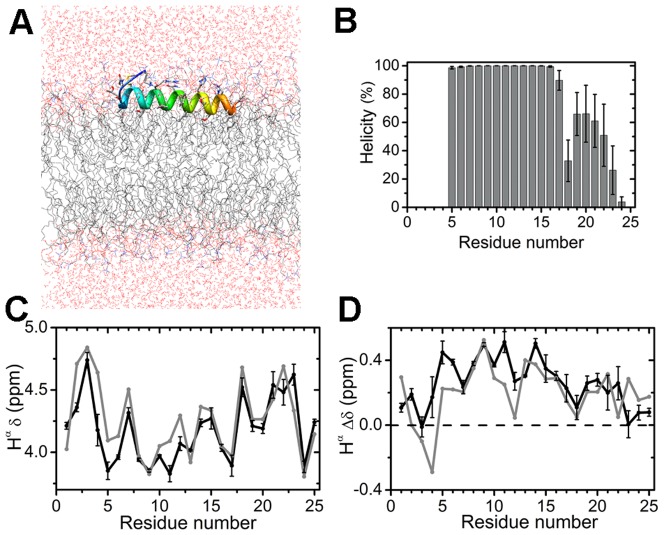
Simulations of membrane-bound wild-type hIAPP_1–25_ (+3). (A) Initial position and orientation of the peptide in the membrane. All of the biomolecular images in this work were generated using the Chimera [71] package. The peptide is rendered as a ribbon with its N-terminus in blue and C-terminus in orange. The water and membrane molecules are shown by the wire model. The coloring scheme for the atoms of water and membrane are: red (O atom), blue (N atom), grey (C atom), orange (P atom) and white (H atom). The scheme is used in all of the following figures. (B) Helicity. (C) H^α^ chemical shifts. The black line denotes the simulation result and the grey line represents the experiment data [12]. (D) Simulated (black line) and experimental (gray line) H^α^ secondary shifts. The average values and the error bars in (B)–(D) were calculated over five trajectories.

After the IAPP peptides were orientated with respect to the membrane surface, the peptides were immersed into the equilibrated DOPC/DOPS bilayers using the g_membed module in GROMACS [45]. This module allows one to insert a protein/peptide into an equilibrated lipid bilayer and keeps the membrane close to its equilibrium state after the embedding. After the insertion, 2 DOPC molecules in each IAPP-membrane system were removed to optimally accommodate the peptide. Three Cl^−^ atoms for hIAPP(+3) and four Cl^−^ atoms for hIAPP(+4) were added, respectively, to neutralize the systems. We used 53A6 GROMOS force field parameter set for the peptides [46]. The peptide-membrane systems were subjected to the energy minimization with the same protocol as that used for the DOPC/DOPS membrane. The systems were then equilibrated in the NVT ensemble for 50 ps followed by an additional 1-ns MD run in the NPT ensemble. The MD simulation protocol was the same as that used for the membrane bilayer. A summary of the simulations is given in Table 1. An illustration of the initial position and orientation of the peptide in the membrane is shown in Figure 1A. We monitored the contacts between the lipid head groups and the protein as a function of simulation time (Figure S1).

**Table 1:** Summary of the simulations.

System	Lipids	Atom (#)	Simulation time^a^ (ns)
DOPC/DOPS	88 DOPC : 40 DOPS	23656	1×50
DOPC/DOPS+hIAPP_1–25_(+3)	86 DOPC : 40 DOPS	23726	5×100
DOPC/DOPS+hIAPP_1–25_ (+3) (reversed orientation)	86 DOPC : 40 DOPS	23723	1×100
DOPC/DOPS+S20G hIAPP_1–25_(+3)	86 DOPC : 40 DOPS	23717	5×100
DOPS+hIAPP (+3)	126 DOPS	24349	1×100
DOPC/DOPS+hIAPP_1–25_ (+4)	86 DOPC : 40 DOPS	23725	1×100

a The multiple trajectories were carried out from the same initial structure, but different initial velocities were assigned.

### Analysis

The immersion depth, D_im_, of a residue is defined as the distance between the z component of the geometric center of a side chain to the z component of the average position of the phosphorus atoms at the peptide-membrane interface. The peptide-membrane interface is the side of the bilayer where the peptide was immersed. The g_dist module in GROMACS was used to calculate the distance. The <D_im_> is the average D_im_ over the simulations. The do_dssp and g_hbond modules of GROMACS were used for the secondary structure assignments and hydrogen bond calculations, respectively. A hydrogen bond is considered to be present if the corresponding hydrogen donor and acceptor are within 3.5 Å and the angle of acceptor - donor - hydrogen is less than 30°. The helicity of a residue is defined as the percentage of occurrence of helical conformations of this residue within an interested simulation time period. The predicted 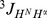 coupling constant was calculated using the Karplus equation [47]:



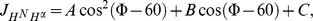
where Φ is the backbone dihedral angle of a particular residue. The A, B, and C parameters are 6.51, −1.76, and 1.60, respectively [48]. The average 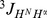 values of the last 50-ns trajectory were used to plot the sequence profile of 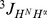. The chemical shifts were predicted using SHIFTX (version 1.1) [49]. The random coil values were from reference [50]. In the analysis of side-chain contacts, two side chains are defined to be in contact if the distance between any of their side-chain heavy atoms (C^α^ atom for Gly) is less than 4.5 Å. The area per lipid and the lipid bilayer thickness were calculated using the toolkit GridMAT-MD [51]. The order parameters (S_cd_) were calculated using the g_order module in GROMACS. The three-dimensional radial distribution function (RDF) of phosphate groups of DOPC and DOPS relative to oxygen atoms of water were calculated using the g_rdf module in GROMACS. The number of water molecules per phosphate group is the cumulative sum derived from the analysis of RDF at the separation distance of 3 Å. The plot of RDF for DOPC/DOPS bilayer is shown in Figure S5. The last 50-ns trajectories were used for the analyses of peptide-membrane systems.

## Results and Discussion

### Wild-type hIAPP_1–25_ (+3 Charge State) in Membrane

We collected five MD trajectories of the wild type of 100 ns each. The helicity is shown in Figure 1B. Residues 5–17 exhibit high helicity, while His18 appears as a helical disruption site. The residues succeeding His18 show lower helicity than those that precedes it. Helical distortion at His18 has been observed under a variety of experimental conditions, though it is usually described as a kink, for example, hIAPP in the sodium dodecyl sulfate (SDS) micelle [12], hIAPP in the helix-inducing solution [52], and IAPP fused to maltose binding protein [53]. Overall, the helicity observed in our simulation is consistent with the result of helical periodicity of EPR (Figure 3A of Ref. [26]), where residue 9 to 22 were identified as a helical region. The EPR data do not include residues 1, 3, and 5–7. We also calculated the H^α^ chemical shifts and the secondary shifts (Figure 1C–D), which are shown experimentally to be sensitive to secondary structures [54]. We reproduced the experimental data [12] well for most of the residues except the N-terminal region and residues 11–12. The discrepancies may be due to the difference in the experimental and simulation conditions: SDS micelles were used experimentally while we simulated DOPC/DOPS bilayers. Generally, both experiments and simulations show that an ordered helical structure is present from residue 5 to residue 17 and a less ordered or a dynamic helix is present in the region after residue 18.

**Figure 2:**
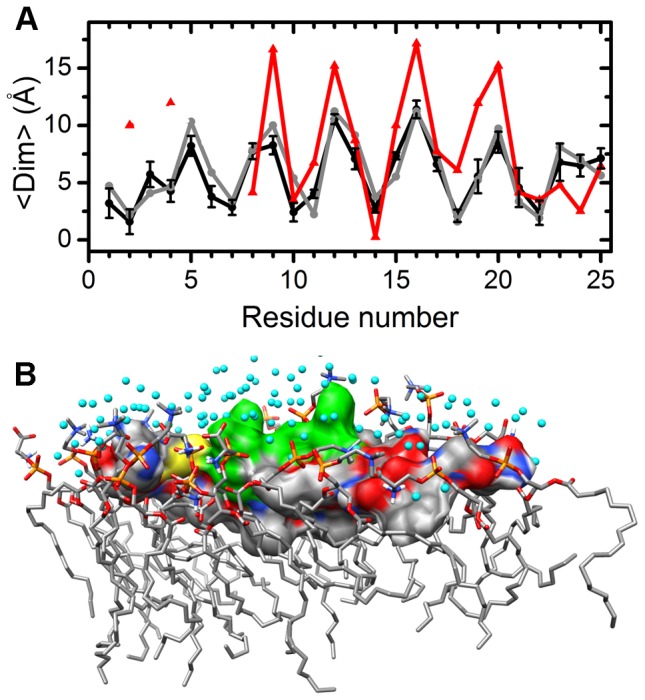
Relative position and orientation of the peptide in the membrane. (A) Mean immersion depth (<Dim>) of each residue. The mean (black line) is calculated from the five simulations and its standard deviation is shown as an error bar. For comparison, the immersion depth of the initial structure (grey line) and the experimental results [26] (red line/symbol) are also shown. The experimental data are not available for residue 1, 3, 5, 6, and 7. Note that the full length (37 residues) IAPP was used in the experiment. The simulation data may be influenced by the truncation at the C-terminus. (B) Wild-type hIAPP_1–25_ (+3) and its nearby molecules (within 4.5 Å) at 100 ns in one of the trajectories. The peptide is represented by the surface model. The solvent exposing residues, Gln10, Arg11, Asn14 and His18, are in green. Other residues are colored according to atom types: sulfur is in yellow and other atoms are colored using the same scheme as Fig. 1. The cyan balls represent the oxygen atoms of water molecules. The lipid molecules are rendered by the stick model.

To investigate the change in peptide orientation during the simulation, the mean of the immersion depth (<D_im_>) of each residue and its standard deviation during the simulation are calculated. As shown in Figure 2A, one side of the helix with Thr9, Leu12, Leu16, and Ser20 is buried inside the membrane, while the other side of the helix with Gln10, Asn14 and His18 is exposed to the solvent, consistent with the initial orientation. This indicates that the relative orientation of the peptide with respect to the membrane surface remains unchanged during the simulations. Figure 2B depicts one snapshot. Compared to the experimental accessibility analysis [26], the overall trend of the simulation data is in line with the experimental result even though the numerical values are different. This is because different distances are measured in the experiment and simulation: the distance from the center of each spin labeling group to the membrane-water interface is detected by the experiment, while we calculate the distance between the geometric center of each side chain and the interface. We also ran a simulation starting from the opposite orientation with Thr9, Leu12, Leu16, and Ser20 exposing to the solvent and Gln10, Asn14 and His18 buried inside the membrane. We find that the peptide turns around quickly, making residue 12 and 16 immerse into the membrane and residue 10 and 14 expose to the solvent (Figure S2). Therefore, the parallel orientation with Gln10, Asn14, and His18 on the solvent-exposing side is favored.

### Membrane-bound hIAPP_1–25_(+3) S20G Mutant

S20G is a fatal mutation that accelerates amyloid formation [6], [7]. Combining the circular dichroism, Fourier transform infrared spectroscopy, transmission electron microscope, and dye binding results, Liu *et al.* concluded that the dominant structure in the fibrils formed by the hIAPP S20G fragment (11–25) is parallel β-sheet, while the wild-type hIAPP_11–25_ forms α-helix-rich assemblies [44]. The authors suggested that S20G facilitates the α-helix to β-sheet transition in the process of aggregation [44]. However, their results of H^α^ secondary chemical shifts and rotating-frame Overhauser effect do not reveal any difference between the wild-type hIAPP_11–25_ and its S20G mutant in the monomeric state in aqueous solution; both peptides show transient α-helical conformations. How does the single-point mutation promote β-sheet-rich aggregation? We investigate this issue by comparing the structural features of the wild type and the mutant in the membrane environment.

**Figure 3:**
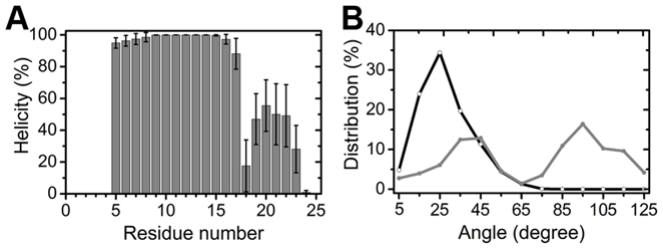
Simulations of hIAPP_1–25_(+3) S20G mutant. (A) Helicity. The average value and the error bar were calculated over five trajectories. (B) Interhelical angle distribution. The angle is defined as that between the vector from the C^α^ of Ala13 to the C^α^ of Val17 and the vector from the C^α^ of Ser19 to the C^α^ of Phe23. The black line denotes the wild type and the gray line is for the S20G mutant. Every 10° is a bin and the midpoint of each bin is represented by a symbol.

Like the wild type, the whole helix is separated into an ordered helix (residues 5–17) and a less ordered helix (residues 19–23) by the disruption site at His18 (Figure 3A). Unlike the wild type, we find that in some mutant conformations the two helices adopt a roughly perpendicular orientation, which resembles an L-shape that bends at His18. To quantitatively describe the orientation, we calculated the angle formed between the vector from the C^α^ of Ala13 to the C^α^ of Val17 and the vector from the C^α^ of Ser19 to the C^α^ of Phe23, which is one helical turn preceding and succeeding His18, respectively. The distribution of the angle is shown in Figure 3B. For the wild type, the mean of the interhelical angle is near 20°, while the S20G mutant shows two peaks: one is at around 40° and the other is near 90°. Using the interhelical angle as a “clustering” criterion, we consider that the wild type has only one conformational family (with a small interhelical angle), while the mutant has two families, the one with a small interhelical angle and the one of the L-shaped motif (interhelical angle ≅ 90°).

**Figure 4:**
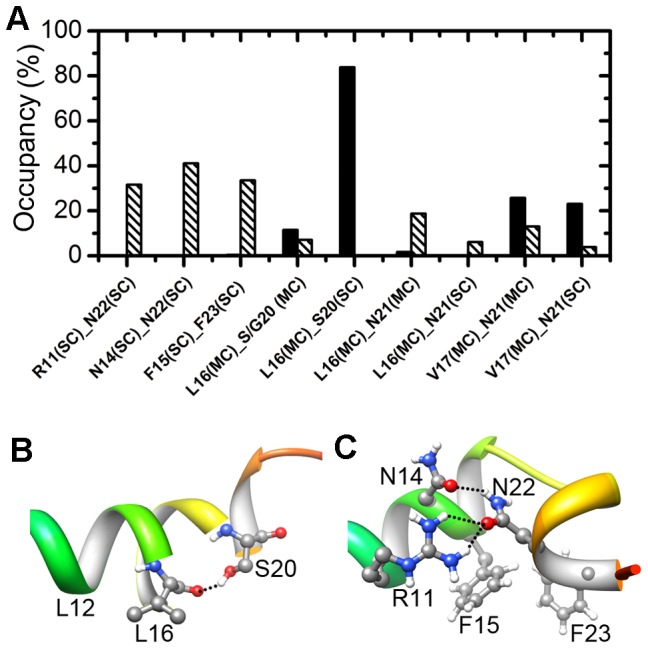
Residue contacts: implications for the L-shaped structure. (A) Occupancy of hydrogen bonds and hydrophobic side-chain contacts of the wild type (black) and S20G mutant (striped). MC and SC denote main-chain and side-chain, respectively. F15(SC)–F23(SC) is the only one hydrophobic contact. All of the other contacts are hydrogen bonds. (B) Hydrogen bonding between the backbone of Leu16 and the side chain of Ser20 in a wild type conformation. (C) Interhelical contacts in a L-shaped structure of S20G mutant. The dotted lines denote the hydrogen bonds.

What interactions obstruct the wild type to adopt the L-shaped motif? To answer this question, we compare the residue-residue contacts in the wild type and the mutant and present the main differences in Figure 4A. For the wild type, the main-chain-side-chain hydrogen bonding between Leu16 and Ser20 is prevalent, present in 83.8% of the conformations. It was reported that the hydrogen bonding between the side chains of serine or threonine and the i-3 or i-4 main-chain carbonyl group is common for membrane-bound proteins [55]. Although the long helix (residues 5–17) is separated from the short helix (residues 19–22) by the helical break at His18, this main-chain-side-chain hydrogen bond imposes a geometric restraint on the helices, leading to a small interhelical angle. When the S20G mutation demolishes this hydrogen bonding, other hydrogen bonds, such as those between Leu16 and Asn21 as well as those between Val17 and Asn21 (Figure 4A), are not present frequently enough to impose the restraint to the same extent. Further, the side-chain-side-chain contacts among residues Arg11, Asn14, Asn22, Phe15, and Phe23 help to stabilize the L-shaped motif in S20G. The main-chain-side-chain hydrogen bonding between Leu16 and Ser20 in the wild type and the side-chain contacts of S20G mutant that stabilize the L-shaped structure are depicted in Figure 4B and Figure 4C, respectively.

**Figure 5:**
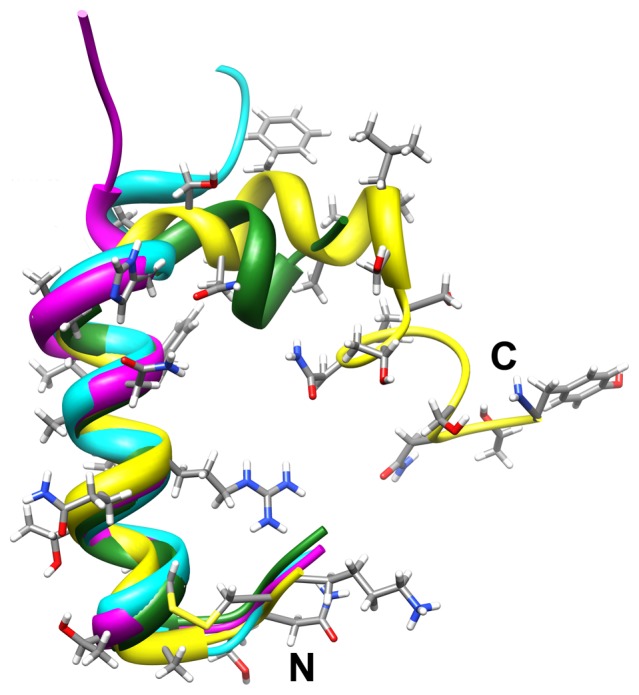
Superimposition of the representative conformations of wild type hIAPP_1–25_ and the S20G mutant with the NMR structure. The NMR structure of hIAPP_1–37_ (+3) (PDB ID: 2L86, model 1) is in yellow [12]. The side chains of the NMR structure are shown using the stick model. The representative structure of the wild type hIAPP_1–25_ is in cyan. The representative structures of S20G mutant are in magenta with the interhelical angle ≅40° and green with the interhelical angle ≅90° (L-shaped motif), respectively.

The L-shaped conformation that we observed in the simulation of the S20G mutant closely resembles the helix-kink-helix motif of the wild-type hIAPP (+3) in SDS micelles at pH 7.3 detected by high-resolution NMR [12] (Figure 5). Their structure reveals two major helices, residues 7–17 and residues 21–28, separated by a short flexible region and the interhelical angle is 85°. We chose one conformation from the 90-degree peak region of Figure 3B as the representative L-shaped conformation. The C^α^ root-mean-square deviation of the ordered helix (residues 5–17) between the representative conformation and the NMR structure (model 1) is less than 1 Å. However, no interhelical contact is present in the NMR structure, while the simulated structure shows the side-chain contacts among Arg11, Asn14, and Asn22 as well as that between Phe15 and Phe23. We searched the helix-kink-helix motif in the protein data bank using PDBeFold [56] and find that the similar structures are stabilized by intramolecular hydrophobic contacts (Figure S3). We suspect that the small size of the SDS micelle happens to stabilize the L-shaped conformation without the intramolecular contacts. We also chose one S20G conformation from the 40-degree peak region (of Figure 3B) and a wild-type conformation from the wild-type 20-degree peak region (of Figure 3B) as representative conformations. Figure 5 also shows the superimposition of these two representative conformations with the NMR structure. None of the representative conformations shows large root-mean-square deviation in the ordered helical region. We speculate that the wild-type hIAPP samples the helix-kink-helix conformation by chance when both the main-chain-main-chain and main-chain-side-chain hydrogen bonds between Leu16 and Ser20 are lost. Indeed, we observed that the simultaneous loss of both hydrogen bonds coincides with the large fluctuations in backbone dihedral angles near His18 in one trajectory (Figure S4). However, for wild type the search for the L-shaped motif (in the lag phase) is likely to occur in a longer timescale than hundreds of nanoseconds.

### Implications of the L-shaped structure in fibril formation

Wild-type hIAPP has lag time of hundreds of seconds, while S20G hIAPP has no observable lag phase [8]. The sequence effect on the propensity of amyloid formation of IAPP variants and other amyloidogenic peptides was attributed to the entropic barrier and the differences in the free energy landscapes [57], [58], [59]. The L-shaped structure observed in our simulation resembles the overall shape of the peptide in the fibril state. The hinge region around residue 18 corresponds to the beginning of the center loop in the β-hairpin structure of IAPP fibrils [60], of which two β-strand segments (residues 8–17 and residues 28–37) are parallel to each other, and the loop region (residues 18–27) is almost perpendicular to the two strands. Recent EPR data and computational refinement show a slightly different model [61]. The conformational preorganization of the S20G mutant monomers into the overall fibril topology would reduce the cost of conformational search in aggregation. Indeed, the L-shaped structure coincides with the faster rate of aggregation in experiments [62]. A high resolution NMR experiment detects that in the presence of 10 mM of Zn^2+^ ion hIAPP adopts a similar L-shaped conformation [62]. The lag time of amyloid formation under this Zn^2+^ concentration is reduced by half [62]. In the study of hIAPP aggregation using two-dimensional IR spectroscopy and isotope labeling [63], it was found that the residues that initially develop in-register are in the ordered loop region of the fibrils. Our simulation suggests that the L-shaped motif in the monomeric state may be the fast route to this in-register form in the early-stage of aggregation. It is possible for the wild-type to sample this L-shaped conformation, too. Yet, its sampling is most likely to be hindered by the backbone rigidity imposed by hydrogen bonding (Leu16 main chain to Ser20 side chain).

### Further validation of the simulations and the force field

Although the lipid parameters were originally developed for the GROMOS87 force field, we validate the combination of the lipid force field with the 53A6 GROMOS protein parameters against the experimental data as shown above, such as the immersion depth and chemical shifts. To carry out further validations, we performed 100-ns MD simulation of pure DOPS with embedded hIAPP_1–25_ (+3) using the same protocol as that of DOPC/DOPS and peptide system. We calculated the basic structural properties of the lipids that describe the “global” equilibrium as listed in Table 2. The lipid packing is measured by the area per lipid molecule, the thickness of the bilayer, and the order parameter of acyl chain (S_cd_). The thickness of the bilayer corresponds to the average phosphorus-phosphorus distance from the top to the bottom leaflets. S_cd_ measures the alignment of the acyl carbons relative to the bilayer normal. As shown in Table 2, the presence of the peptide has small effect on the average “global” equilibrium parameters. The difference between the pure lipids and the protein/lipid system observed in our simulation is comparable to those observed using other force fields [64], [65]. For comparison, the same properties and the hydration of lipids for DOPC/DOPS bilayer without IAPP were also present in Table 2. The hydration of DOPC and DOPS of the mixed lipids is slightly different from that in the pure lipids.

**Table 2:** Structural properties of the lipids.

Properties^a^	Our work		Previous simulation^b^		Experiment^c^	
	DOPC/DOPS	DOPS with hIAPP	DOPC	DOPS	DOPC	DOPS
A_L_ (Å^2^)	67.43±0.17	64.57±0.24	70.55±0.12	63.31±0.08	72.5	64
D (Å)	37.36±0.22	38.5±0.26	35.8±0.2	39.0±0.2	36.9	39
<S_cd_>	0.11±0.04 (DOPC) 0.13±0.04 (DOPS)	0.14±0.04	0.10±0.04	0.14±0.05	0.14	0.15
# of H_2_O molecules	2.88 (DOPC)3.71 (DOPS)	3.47	2.62	3.80		

a The properties are area per lipid (A_L_), thickness (D), the average value of the order parameter of acyl chain (<S_cd_>), which is averaged over the last 50-ns snapshots and the carbon atoms in the chain, and the number of water molecules per lipid phosphate group. The properties for DOPC/DOPS bilayer without IAPP were calculated from the last 10-ns trajectory.

b The previous simulation data are from Ref. [35].

c The experimental value is obtained from the X-ray diffraction data [72].

**Figure 6:**
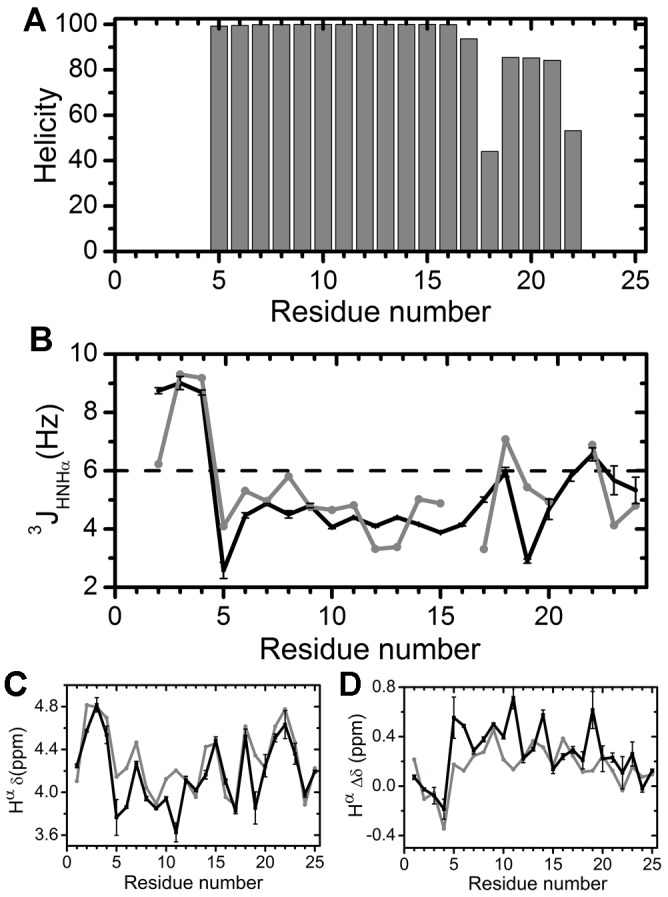
Simulation of hIAPP_1–25_(+4). (A) Helicity. (B) 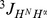 coupling of residues 2–24. No experimental data are available for residue 16 and 21. (C) H^α^ chemical shifts. (D) H^α^ secondary shifts. In (B)–(D), the black lines are for the simulation and the grey lines are for the experimental results [13]. The standard deviation of the average was estimated from the five 10-ns windows in the last 50-ns trajectory.

In order to compare with more experimental data, we carried out one 100-ns MD simulation of hIAPP_1–25_ (+4) in membrane with His18 doubly protonated. Like hIAPP_1–25_(+3), His18 (Figure 6A) shows lower helicity than the helical residues that precede it and succeed it. We predicted the 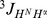 coupling (Figure 6B) to quantitatively compare the simulation results with the experiment data of the hIAPP peptide bound to SDS micelle under an acidic pH [13]. As shown in Figure 6B, in general we reproduce the experimental 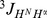 coupling constant well. For example, residues 5–17 show the 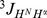 values below 6 Hz, indicating that the Φ angles are roughly in the α-helical range. For comparison, the 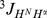 value for residues that adopt a long-lived α-helical structure is 3.9 Hz [66]. And His18 and Asn22 are the only two sites within residues 5–24 that exhibit high 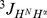 values in both experiment and simulation. However, there are some inconsistencies between the experimental result and the predicted data in the fine structures of the profile, e.g. there is a minimum of 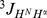 value at Val17 in the experiment, while the minimum moves to Ser19 in our prediction. To complement the 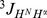 prediction, we also predicted H^α^ chemical shifts and the secondary shifts (Figure 6 C, D). The chemical shifts are in good agreement with the experimental data except Ala5, Arg11, and Ser19 (Figure 6C). Seventy-six percent of our predicted secondary shifts are within ±0.2 ppm of the experimental values (Figure 6D).

The inconsistency between our simulated and the experimental data may be attributed to the difference between the experimental and simulation conditions. Experimentally, hIAPP is bound to SDS micelles. Since micelles are small, their highly curved membranes may introduce bias into the peptide conformation [67]. In addition, the sampling, the force fields [68], and/or the parameterization of the secondary shift prediction method may also affect the simulated data. It is not uncommon in literature that the simulated secondary shifts are consistent with the experimental data in a qualitative fashion, even when enhanced sampling methods are employed [69], [70].

### Summary

In this study we investigate the effect of S20G mutation on the helical structure of hIAPP in the membrane environment using MD simulations. All peptides are initially built into an α-helical structure from residue 5 to residue 25. This conformation is not the global minimum of free energy for all peptides, but it is a good initial structure that is in line with available experimental data [26]. Our simulations are validated against experimental data and simulation results in the literature. The wild type and the mutant are similar in their overall secondary structure: 1) an ordered helical structure runs from residue 5 to residue 17; 2) a helical distortion site is located at His18; 3) a less ordered helical structure ranges from residue 19 to residue 23. However, the fatal mutation of S20G results in a distinct L-shaped structure with a hinge at His18. This L-shaped structure which is stabilized by several side-chain contacts resembles the overall topology of the fibril state. The conformational preorganization of the mutant into the fibril-like topology would reduce the cost of conformational search in the process of aggregation, providing a possible explanation of the fast aggregation rate of the mutant.

## Supporting Information

Figure S1 **Contacts between the lipid head groups and the protein.** The average number of lipid head groups within 4.5 Å of hIAPP_1–25_ (+3) (left column) and S20G hIAPP_1–25_ (right column) as a function of simulation time. If the distance between any heavy atoms of the lipid head group and the protein is less than 4.5 Å, the lipid head group is considered in contact with the protein. The 100-ns simulation trajectory is divided into 100 bins. The average contact number in each bin is plotted. For most of the trajectories, the contact number reaches a plateau region after 50 ns.
(TIF)

Figure S2 **The average immersion depth (<D_im_>) of Gln10, Leu12, Asn14 and Leu16 as a function of simulation time.** The division of bins is the same as Fig. 1S. (A) The initial orientation of peptide was set to be opposite to the experimental results [26]. It can be found that Gln10 and Asn14 move to the solvent-exposing side, while Leu12 and Leu16 immerse deeper into the membrane. (B) The initial orientation and position of the peptide were consistent with the experimental results [26]. These residues basically stay in the initial orientation with respect to the membrane.
(TIF)

Figure S3 **PDBeFold** [**56**] **result of the L-shaped structure in the protein data bank.** The coordinates of (PDB ID) 2L86 model 1 was used as query. The structure of dimerization domain (1–33) of HNF-1alpha (PDB ID: 1JB6) shows that the intramolecular hydrophobic interaction stabilizes its L-shaped structure.
(TIF)

Figure S4 **Hydrogen bonds between Leu16 and Ser20 and the fluctuation of dihedral angles near His18 of hIAPP_1–25_(+3).** (A) Occupancy of hydrogen bonds. The grey line denotes the backbone hydrogen bond between Ser20 and Leu16 and the black line represents the hydrogen bond between the main chain of Leu16 and the side chain of Ser20. The 100-ns simulation trajectory is divided into 100 bins. The percentage of the occurrence of each hydrogen bond within a bin is plotted. (B) Fluctuation of backbone dihedral angles of residues 18–20 in each bin. The grey shadow highlights the region where both the main-chain-main-chain and the main-chain-side-chain hydrogen bonds are lost.
(TIF)

Figure S5 **Three-dimensional radial distribution function (RDF) of phosphate groups of DOPC and DOPS of the DOPC/DOPS mixed bilayer without IAPP relative to oxygen atoms of water.** The last 10-ns trajectory was used for the analysis.
(TIF)
