# Supplementary figures and images for: Conformations of Islet Amyloid Polypeptide Monomers in a Membrane Environment: Implications for Fibril Formation
Source: PLoS One. 2012 Nov 2;7(11):e47150. doi: 10.1371/journal.pone.0047150 (PMC3487734; doi:10.1371/journal.pone.0047150)

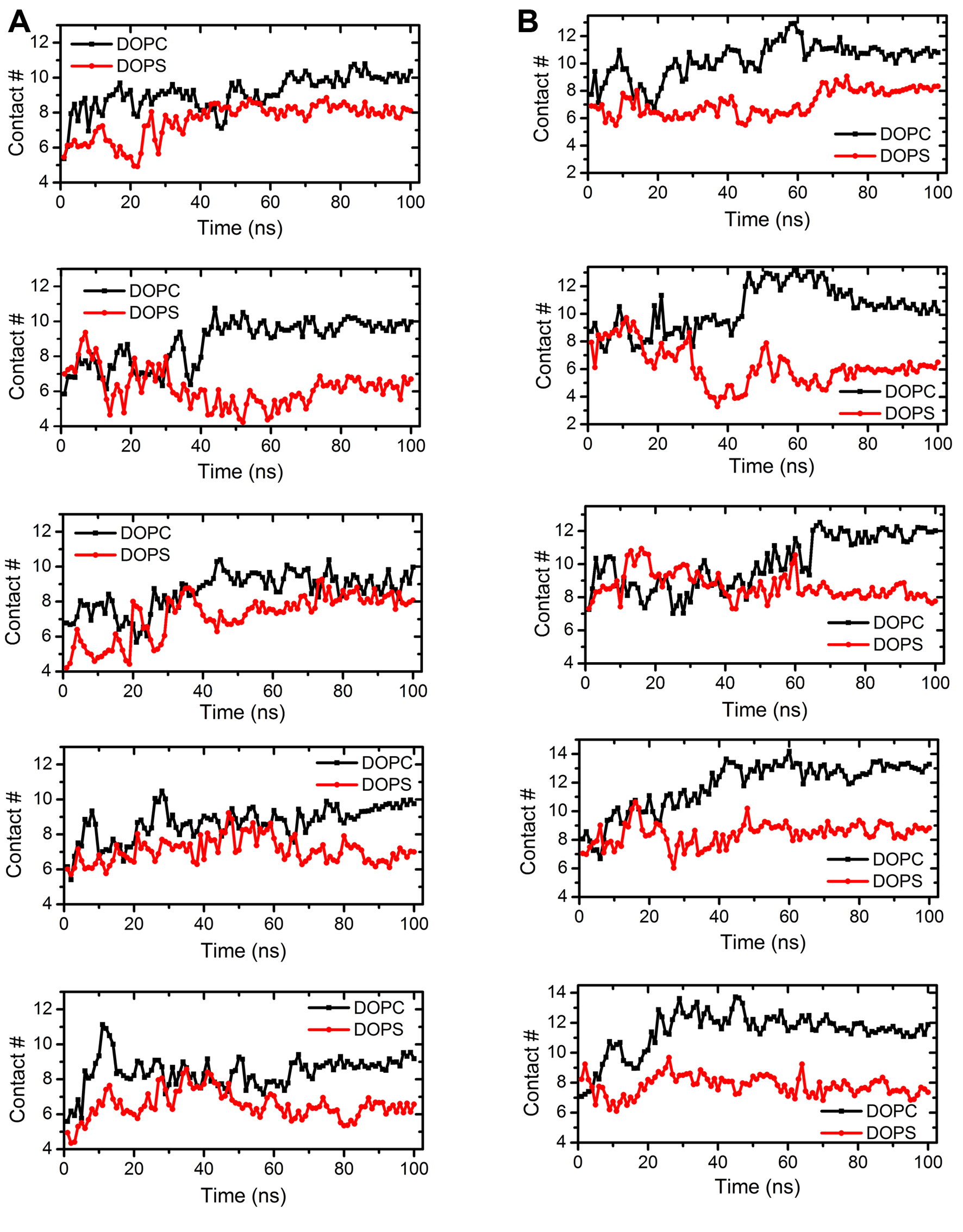

Supplement: Figure S1 — Contacts between the lipid head groups and the protein. The average number of lipid head groups within 4.5 Å of hIAPP1–25 (+3) (left column) and S20G hIAPP1–25 (right column) as a function of simulation time. If the distance between any heavy atoms of the lipid head group and the protein is less than 4.5 Å, the lipid head group is considered in contact with the protein. The 100-ns simulation trajectory is divided into 100 bins. The average contact number in each bin is plotted. For most of the trajectories, the contact number reaches a plateau region after 50 ns. (TIF) [file pone.0047150.s001.tif]

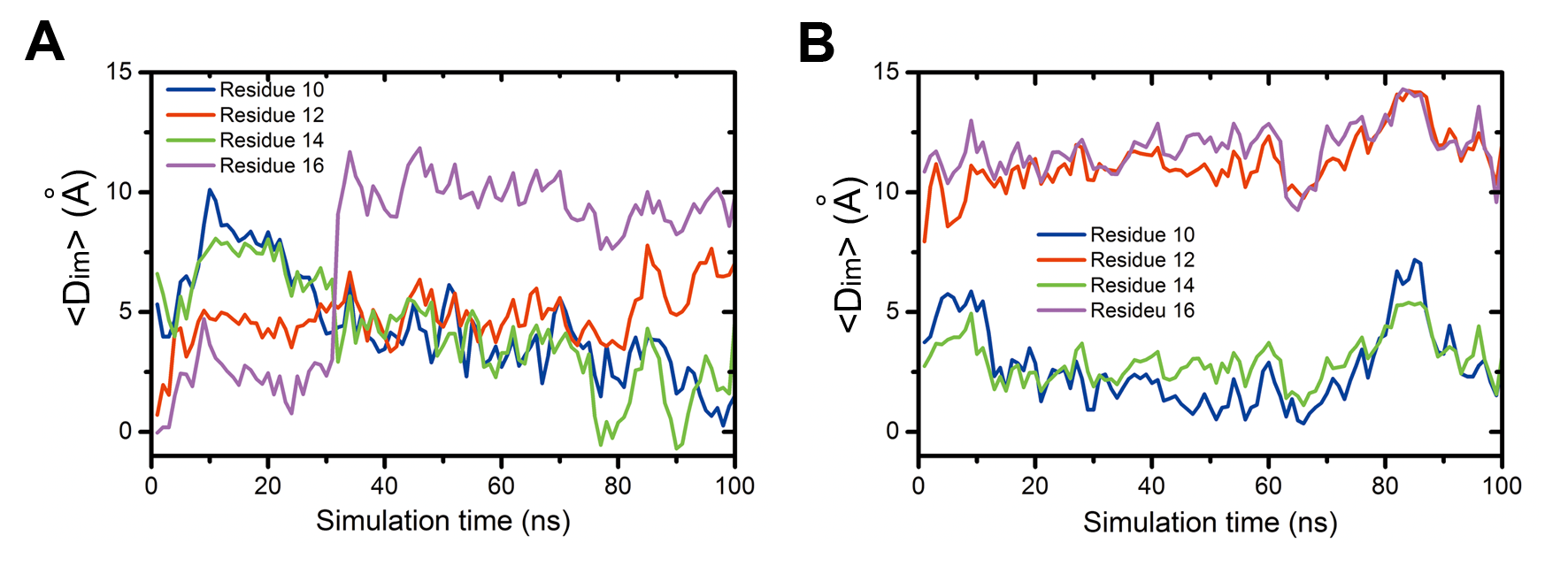

Supplement: Figure S2 — The average immersion depth () of Gln10, Leu12, Asn14 and Leu16 as a function of simulation time. The division of bins is the same as Fig. 1S. (A) The initial orientation of peptide was set to be opposite to the experimental results [26]. It can be found that Gln10 and Asn14 move to the solvent-exposing side, while Leu12 and Leu16 immerse deeper into the membrane. (B) The initial orientation and position of the peptide were consistent with the experimental results [26]. These residues basically stay in the initial orientation with respect to the membrane. (TIF) [file pone.0047150.s002.tif]

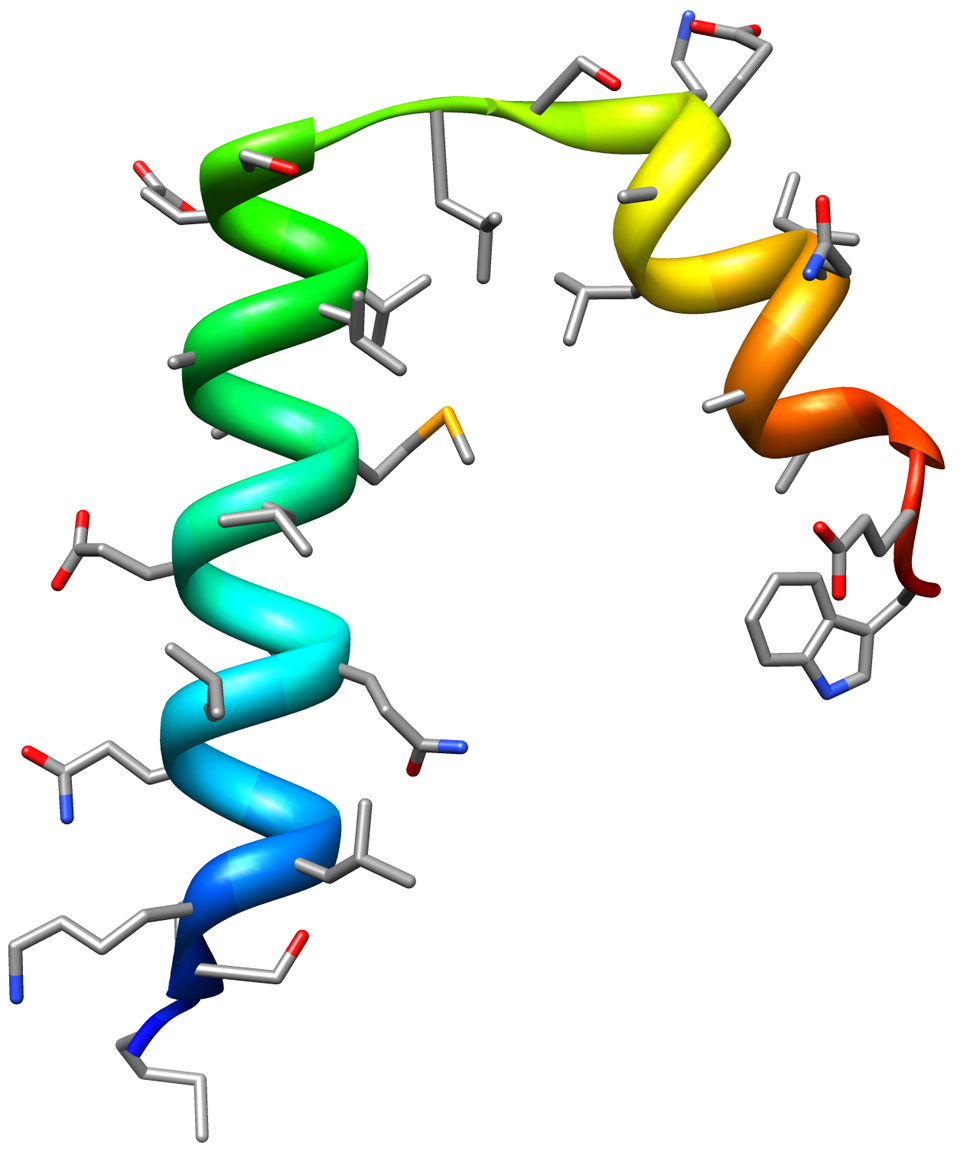

Supplement: Figure S3 — PDBeFold [56] result of the L-shaped structure in the protein data bank. The coordinates of (PDB ID) 2L86 model 1 was used as query. The structure of dimerization domain (1–33) of HNF-1alpha (PDB ID: 1JB6) shows that the intramolecular hydrophobic interaction stabilizes its L-shaped structure. (TIF) [file pone.0047150.s003.tif]

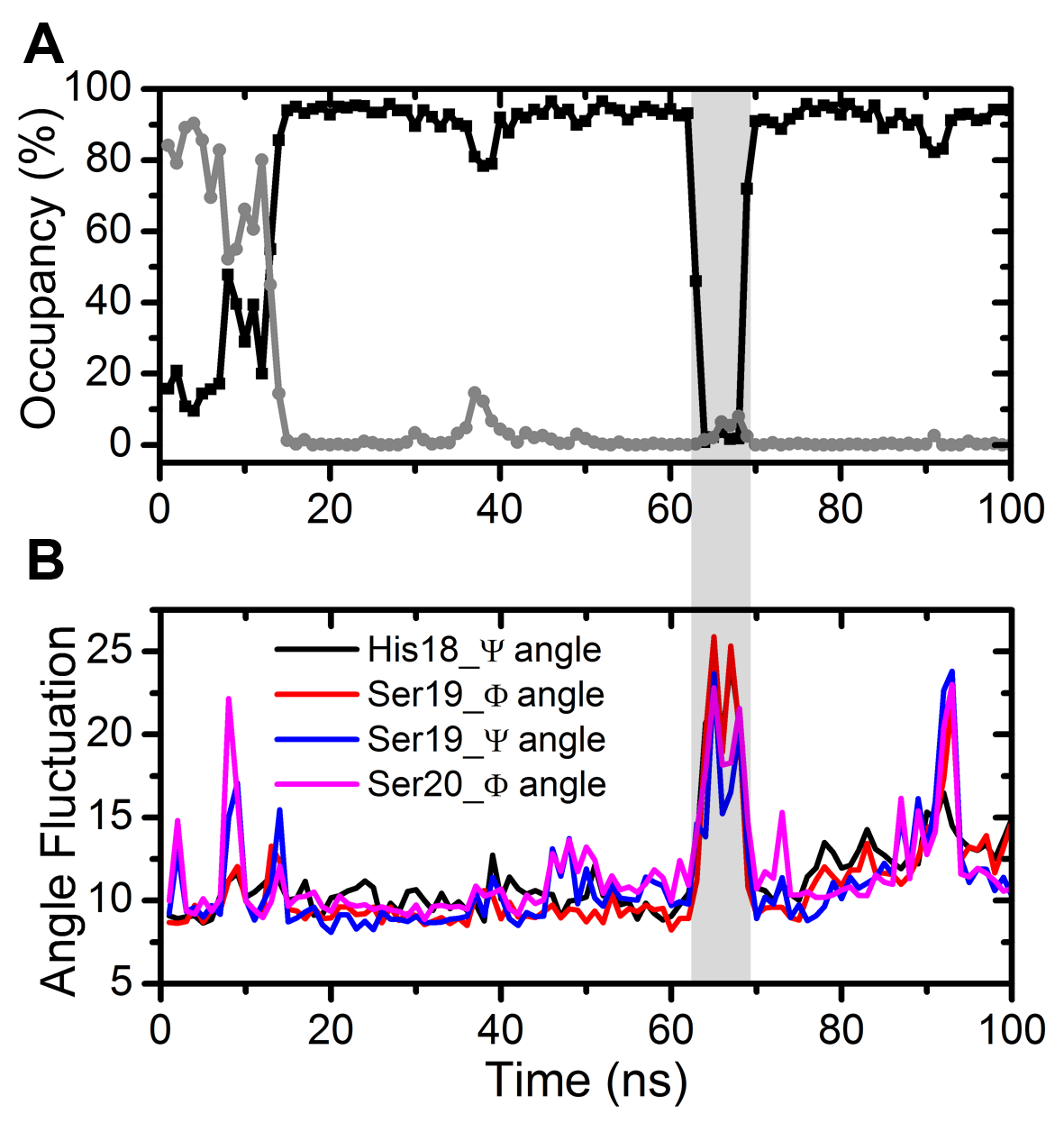

Supplement: Figure S4 — Hydrogen bonds between Leu16 and Ser20 and the fluctuation of dihedral angles near His18 of hIAPP1–25(+3). (A) Occupancy of hydrogen bonds. The grey line denotes the backbone hydrogen bond between Ser20 and Leu16 and the black line represents the hydrogen bond between the main chain of Leu16 and the side chain of Ser20. The 100-ns simulation trajectory is divided into 100 bins. The percentage of the occurrence of each hydrogen bond within a bin is plotted. (B) Fluctuation of backbone dihedral angles of residues 18–20 in each bin. The grey shadow highlights the region where both the main-chain-main-chain and the main-chain-side-chain hydrogen bonds are lost. (TIF) [file pone.0047150.s004.tif]

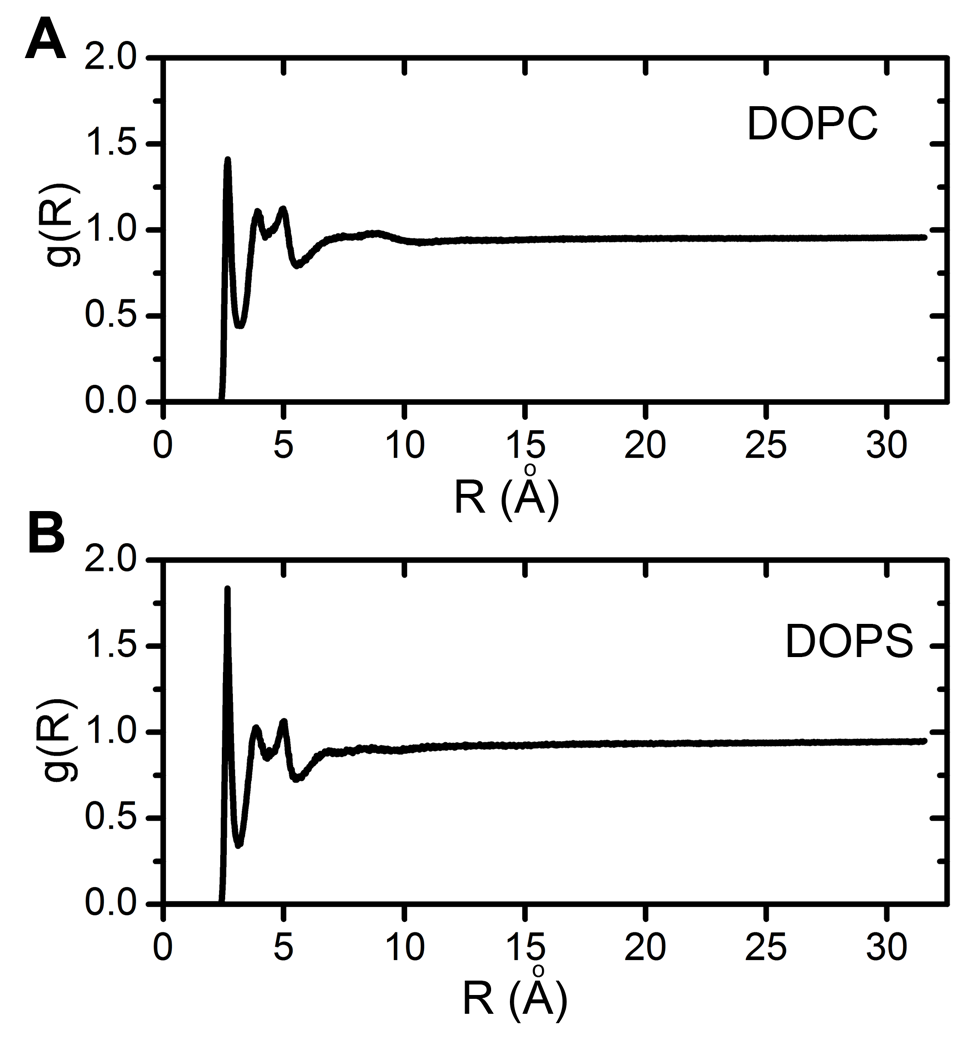

Supplement: Figure S5 — Three-dimensional radial distribution function (RDF) of phosphate groups of DOPC and DOPS of the DOPC/DOPS mixed bilayer without IAPP relative to oxygen atoms of water. The last 10-ns trajectory was used for the analysis. (TIF) [file pone.0047150.s005.tif]
